# Geographic Distribution of Disability Among Older Veterans, United States, 2013–2017

**DOI:** 10.5888/pcd17.190340

**Published:** 2020-05-14

**Authors:** Justin T. McDaniel

**Figure:**
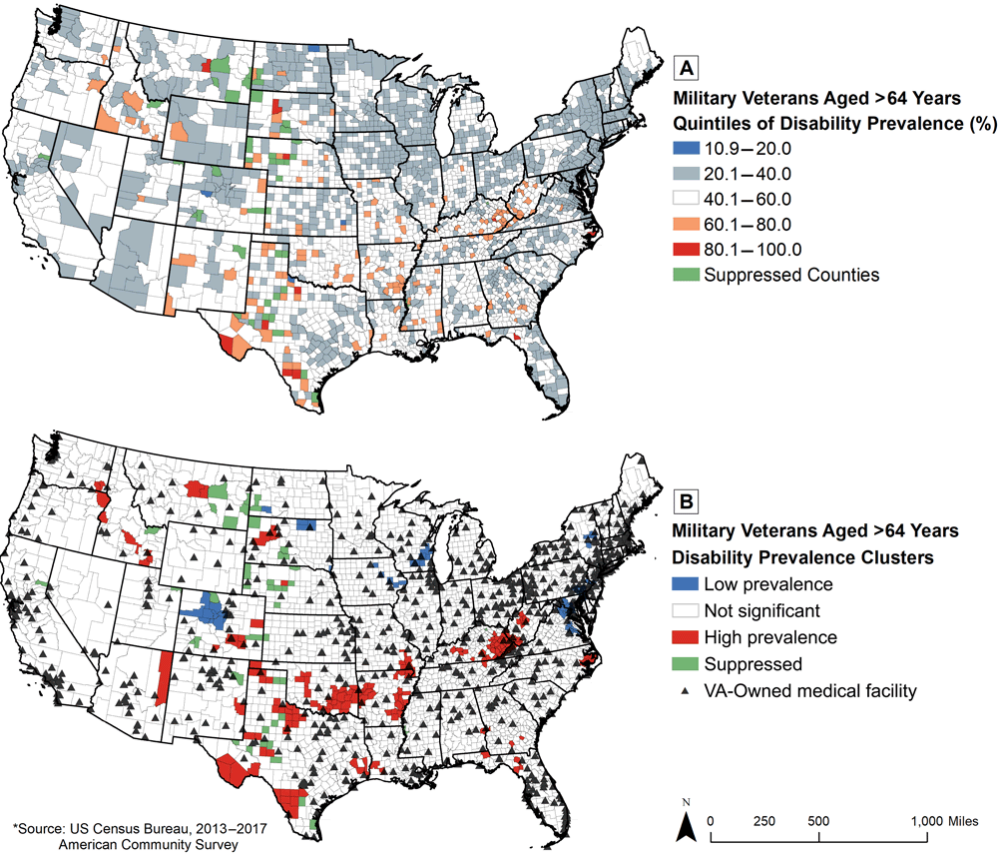
Prevalence of disability among older (aged >64 years) military veterans in US counties. Map A shows county-level prevalence of disability among older US veterans from 2013–2017. Map B shows county clusters of disability prevalence among older US veterans and their location from VA-owned medical facilities. Abbreviation: VA, US Department of Veterans Affairs.

## Background

Disability is a risk factor for chronic disease, including coronary heart disease, cancer, diabetes, obesity, and hypertension (1). Disability prevalence is higher nationally among older (ie, aged >64 y) military veterans (41.4%) than older civilians (36.2%) (2). Because of deployments during military service and exposure to war, veterans are more likely to be wounded and disabled than nonveteran civilians and thus more likely to report a disability, especially in older age (3). Although the overall veteran population is projected to decline by 2024, the percentage of older US veterans is projected to increase from 48.6% to 51.5% between 2014 and 2024 (4). Older veterans are more likely to obtain health care services via the US Department of Veterans Affairs (VA). Because VA hospitals and clinics are sparsely distributed throughout the United States, older veterans’ access to care is limited. An analysis of the geographic distribution of older disabled veterans could improve targeting health care services not only for disability rehabilitation but also for chronic disease prevention and management.

## Data Sources and Map Logistics

I obtained the shapefile for this study from the Integrated Public Use Microdata Series’ National Historic Geographic Information System (5). I retrieved disability data for each county in the contiguous United States (n = 3,108 counties) from the US Census Bureau’s 2013–2017 American Community Survey (6). County-level prevalence of disability among all veterans aged >64 years was calculated by using the number of disabled veterans aged >64 years (N = 3,607,992) as the numerator and number of veterans aged >64 years (N = 9,013,808) as the denominator. Respondents who reported a hearing, vision, cognitive, ambulatory, independent living, or self-care disability were considered disabled (7).

I used ArcGIS version 10.5.1 (Esri) to create 1) a county-level map showing quintiles of disability prevalence among older veterans and 2) a map showing clusters of counties with high and low disability prevalence. Although none of the US Census Bureau’s county-level counts of disabled veterans were suppressed upon retrieval, I suppressed counties (n = 42 counties) that had fewer than 16 older disabled veterans to ensure the stability of rates and subject confidentiality (8). Getis and Ord’s (9) local Gi* was used to identify spatial clusters; neighbors were defined by contiguity of county edges. I used a false discovery rate correction in the analysis to control for multiple testing. I calculated 99% confidence intervals (CIs) for resultant *z* scores from the Gi* analysis to determine spatial clusters. The final layer of this map contains locations of VA-owned medical facilities, which may exclude some leased or contracted community-based outpatient clinics (10).

## Highlights

The national disability prevalence rate for older veterans in the United States from 2013–2017 was 40.0% (99% CI, 40.0%–40.1%). Portions of 28 contiguous states (ie, Alabama, Arkansas, Colorado, Florida, Georgia, Idaho, Illinois, Indiana, Kansas, Kentucky, Louisiana, Mississippi, Missouri, Montana, Nebraska, New Mexico, North Carolina, North Dakota, Oklahoma, Oregon, South Dakota, Tennessee, Texas, Utah, Virginia, Washington, West Virginia, and Wyoming) contained counties with a disability prevalence of 60% or more; 41,476 older veterans resided in these 160 counties, representing 1.2% of all disabled older veterans. Twenty-two (13.3%) of the 165 counties with clusters of high disability prevalence contained a VA-owned medical facility; 18 (35.3%) of the 51 counties with clusters of low disability prevalence contained a VA-owned medical facility.

## Action

In this article, I mapped US Census Bureau data to determine areas of high and low disability prevalence among older US veterans and found that a low percentage of counties with high disability prevalence contained a VA-owned medical facility. This finding provides evidence that health care providers positioned to be responsive to the needs of older military veterans should develop and implement geographically targeted interventions for disability rehabilitation and, possibly, chronic disease prevention or management.

This is the first study to map nationally representative data pertaining to disability among individuals aged >64 years who have served in the US Armed Forces. Two limitations of this study were the exclusion of 42 counties because of unstable rates of disability prevalence and the lack of distinction between types of disability (eg, hearing, vision, cognitive, ambulatory, self-care, or independent living) reported by a particular veteran. Future research should consider more granular levels of analysis (ie, the use of Census tract data), estimate the prevalence of certain disability types (eg, low vision disability prevalence), and determine community-level risk factors (eg, rurality, poverty prevalence) for disability prevalence among older US veterans.

## References

[1] Dixon-Ibarra A , Horner-Johnson W . Disability status as an antecedent to chronic conditions: National Health Interview Survey, 2006-2012. Prev Chronic Dis 2014;11:130251. 10.5888/pcd11.130251 24480632 PMC3917726

[2] Holder KA . Veterans in rural America: 2011–2015. American Community Survey Reports. https://www.census.gov/content/dam/Census/library/publications/2017/acs/acs-36.pdf. Updated January 2017. Accessed October 4, 2019.

[3] Clarke PM , Gregory R , Salomon JA . Long-term disability associated with war-related experience among Vietnam veterans: retrospective cohort study. Med Care 2015;53(5):401–8. 10.1097/MLR.0000000000000336 25768060 PMC4396733

[4] Amaral EFL , Pollard MS , Mendelsohn J , Cefalu M . Current and future demographics of the veteran population, 2014–2024. Popul Rev 2018;57(1):28–60. 10.1353/prv.2018.0002

[5] National Historic Geographic Information System. Download US Census data tables and mapping files. https://www.nhgis.org/. Updated 2019. Accessed December 22, 2019.

[6] United States Census Bureau. American Community Survey data. https://www.census.gov/programs-surveys/acs/data.html. Updated 2019. Accessed October 3, 2019.

[7] United States Census Bureau. How disability data are collected from the American Community Survey. https://www.census.gov/topics/health/disability/guidance/data-collection-acs.html. Updated 2017. Accessed October 4, 2019.

[8] Centers for Disease Control and Prevention. Suppression of rates and counts. https://www.cdc.gov/cancer/uscs/technical_notes/stat_methods/suppression.htm#1. Updated 2019. Accessed December 23, 2019.

[9] Getis A , Ord JK . The analysis of spatial association by use of distance statistics in geographical analysis 1992;24(3):189-206.

[10] United States Department of Homeland Security. Veterans Health Administration medical facilities. https://hifld-geoplatform.opendata.arcgis.com/datasets/f11d7d153bfb408f85bd029b2dac9298_0. Updated 2017. Accessed December 23, 2019.

